# Vascular endothelial growth factor and its receptors regulation in gestational diabetes mellitus and eclampsia

**DOI:** 10.1186/s12967-022-03603-4

**Published:** 2022-09-05

**Authors:** Alayi Bolatai, Yujing He, Na Wu

**Affiliations:** 1 Student Affairs Department, Shengjing Hospital of China Medical University, Shenyang, Liaoning Province People’s Republic of China; 2 Department of Endocrinology, Shengjing Hospital of China Medical University, Shenyang, 110004 People’s Republic of China; 3 Medical Services Section, Shengjing Hospital of China Medical University, Shenyang, 110004 People’s Republic of China

**Keywords:** Vascular endothelial growth factor (VEGF), Vascular endothelial growth factor receptor (VEGF-R), sFlt-1, KD2, Gestational diabetes mellitus (GDM), Eclampsia, Preeclampsia (pe)

## Abstract

**Background:**

An imbalance in the expression of vascular endothelial growth factor (VEGF) and its receptor (VEGF-R) during pregnancy plays an important role in the pathogenesis of gestational diabetes mellitus (GDM) and eclampsia. VEGF and its receptors change during the regulation of blood vessels as a result of risk factors such as familial genetics. These modifications include loss of original balance of serological indicators, upregulation or downregulation of growth factor indicators, and changes in the placenta, kidney, liver and other organs to varying degrees of damage. This has an impact on both the pregnant woman's and the fetus's health.

**Main body:**

This paper summarizes the mechanisms of unbalanced VEGF and receptor expression based on data from relevant literature on GDM and eclampsia. An Imbalance in VEGF and its binding receptor is often associated with the occurrence of multiple pregnancy disorders. In recent years, researchers have focused on the potential role of VEGF and its receptors in the development of GDM and eclampsia.

**Conclusion:**

This paper summarizes the different VEGF subtypes and their binding receptors, as well as mechanisms that cause GDM and eclampsia, in order to provide valuable data to inform monitoring, diagnosis, and prognosis.

## Background

In nature, all endothelial growth factors are glycoproteins, and they form a family with a unique function of regulating the body. The most common types in this family are VEGF-A, VEGF-B and VEGF-165B. The endocrine gland-derived vascular endothelial growth factor VEGF (EG-VEGF) was discovered recently [1]. The most important function of VEGFG is to promote vascular endothelial growth, especially in the bones and embryo [2], VEGF participates in differentiation and growth, including increased vascular permeability, small molecule transport, and anti-apoptosis. Furthermore, it is crucial for the development and construction of blood vessels and lymphatic networks [3]. Normal levels of VEGF promote homeostasis and help in the maintenance of a healthy pregnancy; however, if the balance is disrupted, VEGF concentration increases the probability of other diseases, especially pregnancy complications [4]. VEGF binds to and activates the VEGF receptor (VEGF-R), which is mediated by tyrosine kinase and influences associated physiological changes. VEGF-R proteins (proteoglycans and integrins) can regulate cell physiological properties such as cellular uptake, degradation, and recycling speed [5]. Under VEGF and VEGF-R interaction, blood vessels grow and undergo apoptosis, thereby maintaining a dynamic balance. Pregnancy is accompanied by fetal growth and development, along with changes in several physiological functions. With increasing gestational age, the fetus requires more nutrients, resulting in pregnant mothers absorbing more glucose. Because the renal tubules reabsorption of glucose has limited values, eventually blood glucose levels exceeded the normal range, and the probability of abnormal blood glucose was much higher than in non-pregnant women, with the change of hormone level and islet function of pregnant women, it is easy to develop GDM. During pregnancy, the regulation of maternal blood pressure function decreases, as does persistent vasospasm. At the same time, vascular endothelial damage and activation of inflammatory mechanisms, along with changes in the value of VEGF and other pro-factors, can lead to eclampsia. In eclampsia and gestational diabetes mellitus (GDM), VEGF and VEGF-R values change significantly. As a result, the two diseases always complement each other.

As a result, we can speculate that VEGF and its antagonistic receptor are important related factors. Based on recent GDM and eclampsia data, this article reviews this mechanism,

and mines certain rules for future research. Regulation of the relationship between VEGF and receptor number is important for other diseases related to VEGF and its receptors. For example, some studies have adopted anti-VEGF to further treat retinal diseases caused by diabetes [6]. Appropriate methods can regulate the relative relationship between VEGF and its receptor and reduce the occurrence of pregnancy diseases if drugs acting on specific gene targets or against VEGF are used.

## VEGF and its receptors

### VEGF and its receptor subtypes and functions

VEGF is a cytokine that has a wide range of actions. VEGF-A, VEGF-B, placental growth factor (PLGF), and other types of VEGF interact with their corresponding receptors, promoting vascular growth, changing substances through permeability, and inhibiting tumor development [1]. Within the VEGF family, VEGF-A has a strong relationship with the growth and development of blood vessels and can be induced by single isoform VEGF subtype orVEGF-A164/5 [7, 8].

Exploring the function of VEGF-A can help in understanding its relationship to pregnancy complications. VEGF-A functions as of active regulator on the immune microenvironment, increasing the possibility of graft survival (for example, by regulating the microenvironment of the corneal graft bed [9]. This subtype promotes tumor angiogenesis while also acting as an immunosuppressor throughout the whole body [10]. PIGF is also an important growth factor, and its primary function is to monitor placental function. VEGF165 is highly active in vivo, and VEGF165a, which promotes micro-angiogenesis, and VEGF165b, which inhibits micro-angiogenesis are formed after exon shearing in vivo, and VEGF165b studies have been associated to tumors [11]. VEGF-B has a strongly correlation with islet function and can disrupt islet metabolism by affecting the expression of the endothelial fatty acid transporter [12].

Different types of VEGF can also activate specific receptors. VEGF-A can activate two tyrosine kinase receptors (VEGF-R1 and VEGF-R2) [13, 14], and its receptor values are also affected by VEGF isotypes, indicating that there is mutual feedback between VEGF and its receptors. Furthermore, VEGF-165b, VEGF-B, and VEGF-A are linked to embryonic implantation and placenta formation [4]. Maintaining a constant VEGF value within a certain range can help ensure a healthy pregnancy. However, VEGF imbalance increases the probability of disease development, especially in pregnant women. It is easy to determine gestational hypertension, gestational diabetes, excessive amniotic fluid, severe dystocia, and other complications, for the fetus also has the risk of premature birth, macrosomia, malformation, and other diseases; as a result, VEGF is a very important pregnancy regulator. And the renin-angiotensin aldosterone system (RAAS) regulation mechanism is accompanied by the change in VEGF [15], Blood pressure is out of balance, especially during pregnancy, and VEGF and other growth factors are increased, increasing the likelihood of pregnancy complications.

Combination of VEGF and its receptors via the VEGF/VEGFR signal pathway, tyrosine kinase receptor family composition in three areas: the area across the membrane, seven immunoglobulin sample structure composed of extracellular domain area and tyrosine kinase intracellular area [16], VEGF combined with a specific area of the corresponding receptors [17], after combining the phosphorylation reaction, induction of different reaction.

VEGF-related receptors also regulate vascular growth similar to VEGF, but in essence are VEGF inhibitors. Most antagonist receptors mainly regulate normal vascular growth by eliminating VEGF function. The specific mechanisms of antagonistic action have multiple explanations. For example, VEGFR-1 and VEGF-2 can be recognized by VGB-3 (VEGF-A/B antagonist peptide) [18], thus inhibiting the growth of blood vessels. It can be inferred that VEGF-R indirectly regulates vascular growth and differentiation. Moreover, as a receptor tyrosine kinase, VEGF-R can regulate cell absorption, degradation, and recycling functions [19]. This can be used clinically to treat diseases by regulating cell migration or apoptosis. Currently,, relevant studies can use the mutual regulatory characteristics of VEGF and VEGF-R to inhibit the growth and metastasis of tumor cells [18], as well as relieve complications in the fundus and kidney. It is therefore crucial to ensure the stability of VEGF and homeostasis in the body.

### VEGF and pregnancy complications

The mechanism of pregnancy complications is often accompanied by changes in peripheral blood cytokines, and the serological values of diabetic patients are often accompanied by the deposition of various cytokines [20]. Many protein molecules in the body exceed the normal range. The main focus of this review is VEGF and its receptor. In a state of VEGF and receptor imbalance, the probability of pregnancy-related complications is greatly increased. As a result, the health of blood vessels is essential for fetal development and a healthy pregnancy. Maternal vascular function has an effect on both of fetal and maternal life. The ligand of chemokine (CXCL1) plays an important role in promoting angiogenesis [21], and VEGF is an important component in regulating this process. In particular, VEGF-A participates in the regulation of CXCL-1 in blood vessels and decidual angiogenesis. In hypoxia and ischemia, VEGF and its receptors are increased by the ligand of MMP-9 and membrane, mobilizing endothelial progenitor cells to fight inflammation and promote angiogenesis [22]. Therefore, the complications of pregnancy caused by VEGF have aroused people's concern.

In recent years, a study compared two groups of patients with GDM or in good health, and the groups of serological numerical data revealed that patients with GDM had relatively high expression of VEGF serological values and gene polymorphisms [23]. With the higher serological values, it can be inferred that VEGF is a strong correlation factor with GDM. Genetic directions are also relevant, such as the amount of VEGF discrepant expression in the placenta and trophoblasts. Thus, the progression of GDM can be detected. In addition, VEGF can also lead to abnormal fetal blood glucose. Abnormalities in fetal blood glucose values are indirectly predicted by umbilical venous blood VEGF [24]. Fetal growth restriction and giant fetal groups had higher levels of abnormal sFlt-1 expression than the general pregnancy population [25]. Free placental mRNA, free placental DNA gene and other genetic substances also increased. This indicates that changes in VEGF and its soluble receptors are common features of GDM pathogenesis. However, after pregnancy, some data showed a decrease or even recovery of VEGF-related values. Abnormalities in VEGF values were strongly correlated with the pregnancy course. Combining the above, impaired vascular status can be explained by the mechanism of VEGF and its receptors, and VEGF is associated with GDM.

**Fig. 1:**
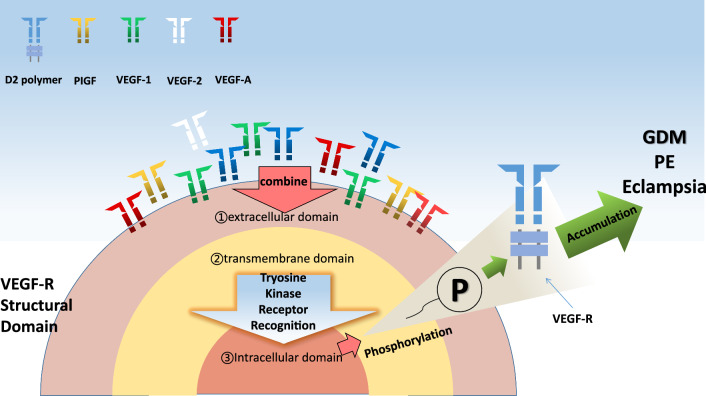
VEGF/VEGFR drives the *signaling tyrosine kinase pathways* of GDM and eclampsia related pathological parameters

Under the accumulation of multiple adverse factors, such as genetics, maternal energy insufficiency, and compensatory vascular growth, the fetus undergoes compensatory increase in levels of VEGF and other promoting factors to maintain balance. In another set of data, we found that sFLT-1 increased in proportion in eclampsia patients, while VEGF, PLGF and s-EGFR decreased [26]. In general, an increase in SFLT-1 will combine with other endothelial factors to inhibit binding, decrease the normal infiltration ability of placental villi and the ability to promote vascular growth, and aggravate vascular activity in the process of eclampsia during pregnancy. As a result, it is easy to see changes in VEGF and its related tyrosine kinase receptors in pregnancy complications, and there are also obvious clinical changes on the basis of vascular injury, oxidative stress and other factors. However, the correlation of VEGF and its receptors with GDM and eclampsia discussed in this paper needs more case data to be demonstrated, so specific relevant mechanisms are shown in Fig. 1. Figure 1 specifically illustrates the process that VEGF binds to the corresponding VEGFR in the process of VEGF/VEGFR tyrosine kinase pathway, gradually evolves into dimer and accumulates to form GDM and eclampsia.

## GDM and VEGF

### Mechanism of GDM development

GDM is one of the most serious complications in pregnancy. Diagnosis is usually based on the first occurrence of diabetes, which is associated with poor blood glucose control during pregnancy. A significant number of women with GDM still have poor blood glucose control or lifelong diabetes after pregnancy. Obesity, a genetic history of diabetes mellitus, and menstrual disorders are all risk factors for the development GDM. Long-term effects of previous risk factors include poor glycemic control in pregnancy, decline in pancreatic islet function, and, ultimately, GDM.

Multiple blood vessels of the mother pass through the paracrine, while autocrine regulatory substances and environmental effects affect the activity of the placenta [27], which then affects fetus. Vascular function is clearly abnormal in the context of inflammation and oxidative stress caused by a blood lipid hormone disorder [28, 29] and various vascular factors affect the normal growth of blood vessels. Genetics also plays an important role in the pathological process here. A number of studies show that individual genes (such as PHLPP1) demonstrate abnormal expression in GDM [30]. GDM patients who do not have significant high-risk lifestyle habits do not usually show obvious signs of disease. Without effective control, both the fetus and the pregnant mother are affected, with outcomes including fetal anemia, high levels of serum erythropoietin, and other abnormal manifestations [31]. In severe cases, GDM greatly reduces fetal quality of life and threatens the mother's life. As a result, detecting VEGF and its receptor is an important monitoring factor for GDM. To that end, it can reduce risk factors and antagonize VEGF, its receptors, and other angiogenic markers to alter the progression of GDM.

### Correlation of GDM and VEGF and its receptors

Data from recent years imply that VEGF is strongly associated with GDM. In some GDM patients with VEGF-A, endothelin and endothelin-1 levels were significantly increased [27]. VEGF-A, endothelin, and G-CSF (granulocyte colony-stimulating factor) have been shown to have a significant positive correlation with endothelin-1. It has been demonstrated that VEGF-A is associated with GDM pathogenesis. The VEGF ratio is positively correlated with intercellular adhesion molecule (ICAM-1) and Advanced Glycation End Products (AGEs) [32, 33]. Abnormal SUCNR1 (succinate receptor) content in umbilical vein blood and associated VEGF gene expression are also increased [34]. Regulation of other factors can influence VEGF-A levels and affect the occurrence of GDM. G-CSF can promote VEGF secretion and thus regulate blood vessel function. ICAM-1 is known to show low expression with AGEs at normal levels. The correlation between ICAM-1 and VEGF ratio expression is significantly higher than that of AGEs. VEGF is associated with impaired inflammatory blood vessel function. The increase in VEGF ratio can be used to detect the expression degree of vascular inflammation in GDM patients; this has definite quantitative significance. The combined effects of multiple factors can be incorporated into GDM detection criteria. As a result, focusing on various angiogenic markers is beneficial. GDM progression and prognosis can be quantified using variables affecting VEGF indicators, such as weight and chorionic vessel quantity [35, 36]. From a microscopic molecular perspective, VEGF also has some exploration value in GDM.

A recent study found that the protein expression of VEGF and PlGF/VEGF-R1 was different compared with that of healthy pregnant women [35]. On this basis, the mRNA and protein content expression of VEGFR-1 (Flt-1) were significantly reduced. VEGFR-2 (KD2) has high mRNA and protein levels [37]. This approach also varies by different production methods. It is evident that VEGF-R1 mRNA and protein are positively associated with VEGF-R2 receptor mRNA but negatively associated with its protein expression. This indicates that the expression of receptors varies. Further research into the relationship between specific genes and expression is needed. Moreover, the mRNA and protein content of Flt-1 are also correlated with the production mode. The content of Flt-1 for cesarean section in GDM patients affects, and it can thus be assumed that changing production methods can affect VEGF levels. Inhibition-related indicators are an important treatment method for GDM. In addition to the difference between microscopic expression and production mode, VEGF receptors are differentially expressed at different sites in GDM patients. Strong staining of VEGF-R1 was detected in GDM patients in both vascular and trophoblast cells. VEGF-R2 and VEGF are only detected in the trophoblast [38]. This indicates that VEGF-R1 has a relatively wider range of action. VEGF was lower than normal in pregnant women with past or present GDM. in a population of patients with previous GDM, VEGF-R1 and VEGF-R2 content also changed accordingly [39]. As a result, pregnancy can affect VEGF and its receptor levels. However, if the blood glucose regulation mechanism is dysfunctional, VEGF serological levels will be far from the normal range. As research has progressed, VEGF and its receptors have also been confirmed to be genetically associated with GDM families. This is supported by the gene polymorphism between VEGF rs2146323 and rs-3025039, as well as the high expression of the PHLPP1 gene in the genotype distribution of GDM patients [30, 40]. The discovery of special GDM genotypes emphasizes the importance of early pregnancy screening. With age, the accumulation of more common factors, such as weight gain, is typical. Therefore, testing genotypes can confirm potential risk early on.

## Eclampsia and VEGF during pregnancy

### Mechanism of eclampsia development during pregnancy

Similar to GDM, eclampsia is another complication associated with VEGF during pregnancy. The continuous progression of the disease is a main concern for eclampsia patients. PE symptoms are more typical. Eclampsia can be confirmed if different degrees of convulsions occur as a result of PE, eclampsia can be confirmed. Higher blood pressure and proteinuria after 20 weeks of pregnancy are also primary characteristics. PE-eclampsia develops gradually as a result of hypertension during pregnancy. Therefore, real-time blood pressure detection is a key factor for health protection during pregnancy. There are many factors that contribute to gestational eclampsia: pre-pregnancy BMI >  = 24 kg/m^2^, smoking during pregnancy, abnormal glucose metabolism, long-term use of insulin, adverse pregnancy history, and chronic hypertension. Once diagnosed, it is important to adjust life and eating habits as soon as possible. Drug treatment, including labetalol, nifedipine or nifedipine sustained release tablets treatment or intravenous medication is also an option, but the use of atenolol and Piperazole is not recommended [41]. PE also contributes to placental dysfunction by similar mechanisms to gestational diabetes. While VEGF, PLGF, hypoxia-inducible factor (HIF-1) and other serological-level growth factors work together regulate the normal growth and development of the uterus and placenta [42].

An imbalance of multiple serological levels can lead to eclampsia during pregnancy and other more severe outcomes. Furthermore, it has been observed in related studies that VEGF imbalance precedes the increase in maternal–fetal complement. Maternal pathogenesis precedes the placenta, driving the progression of PE [43]. Predisposition to PE is usually due to VEGF antagonistic receptor abnormalities as the main manifestation. This receptor is also known as sFlt-1 (soluble fms-like tyrosine kinase-1) and s-Eng (soluble endoglin) [44]. This is one of the direct causes of the accompanying symptomatic proteinuria and hypertension during the course of eclampsia in pregnancy. In conclusion, eclampsia is hidden in the early stage of pregnancy but develops rapidly and seriously in the later stage, and systemic endothelial dysfunction may cause damage to all organs. The liver and kidney function of pregnant women will be further impaired accompanied by the progression of eclampsia. Regulating the condition can reduce the risk value related to pregnancy.

### Association of gestational eclampsia with VEGF and its receptors

VEGF-R imbalance changes more significantly in eclampsia patients than in GDM patients. In a state of increased sFlt-1, free VEGF and PLGF levels were below normal, demonstrating that sFlt-1 antagonizes VEGF in pregnant women with eclampsia [45, 46]. VEGF-R levels are regulated by many factors and affect the promoting or inhibitory effects of vascular growth. Among them, regulation of miR-646 plays an antiangiogenic role through the expression of VEGF-A, and HIF-1[47]. The expression of DNA methylation of VEGF, FLT-1 and KDR changes in PE development [48]. On the other hand, hypomethylation of the VEGF promoter, together with a compensatory upregulation of the corresponding mRNA, can control disease progression and regulate the abnormal blood vessel growth induced by VEGF. The estimated occurrence of PE is correlated with the expression of DNA methylation. Moreover, VEGF expression is also regulated by post-transcriptional modification of micro-RNAs (miRNAs). In particular, for miR-16, significant changes in miR-200c were observed [49]. This shows that the above expressed molecules are related to one another. Serological monitoring and genetic screening of pregnant women can be performed to detect progression of eclampsia prior to PE formation. The gene encoding VEGF rs10434 rs2010963 differs significantly from normal genes [50, 51]. Specific PE-associated genotypes are also suggested. Therefore, the correlation between PE and genetics is useful preparing for or performing genetic screening during the early stages of pregnancy. Understanding the relevant genetic laws can inform disease diagnosis and targeted treatment strategies. VEGF165b increases in healthy pregnant women, with VEGF165b value reduction only observed in PE women at 12 weeks of gestation [52]. This is an important marker for blood vessels in PE. However, the detection time has certain limitations. VEGF, sFlt-1, and PLGF are all important PE factors that contribute to endothelial dysfunction. Pravastatin can inhibit this endothelial functional defect to some extent [53]. Differences in specific detection techniques can play a role in the prevention of PE in early pregnancy.

This may be related to the corresponding test index and the different progression of the disease. Therefore, it is important to adopt effective detection techniques that may better change and highlight the effect of the course of treatment. While VEGF and its associated inhibitory receptors mainly cause pregnancy complications, it has also been implicated in coronavirus disease 2019 (COVID-19). COVID-19, when combined with VEGF imbalance, can increase the probability of developing pregnancy complications [54–56]. Relevant mechanisms are associated with COVID-19 invasion of the placenta, under conditions where VEGF imbalance is already apparent. The blood vessel itself causes pathological damage, and COVID-19 aggravates this process.

## VEGF leads to gestational diabetes and eclampsia

### VEGF and its receptors cause similarities and differences between GDM and eclampsia

**Fig. 2:**
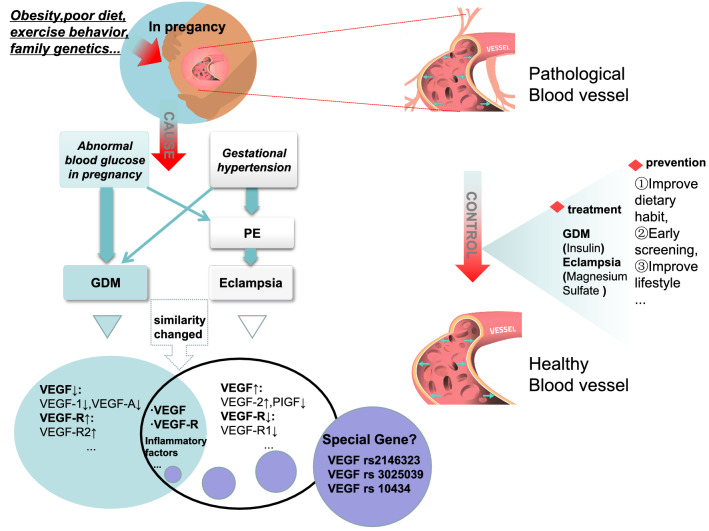
Relationship between GDM, Eclampsia/PE VEGF and its pathogenesis

The relationship between VEGF and its receptor and GDM, PE/ eclampsia can be seen in Table 1. In a group of clinical trials, it can be seen that the VEGF and GFM-PE group are different from normal group, and the expression level of FLT-1 in PE and GDM-PE groups is higher [57]. Therefore, we believe that VEGF and its receptor may be correlated with these two pregnancy complications. Because both VEGF and its receptor change after endothelial injury, which occurs in both GDM and PE diseases, so it can be further inferred that the two diseases can also be associated with each other. Both diseases have similar cumulative risk factors, mainly genetic and lifestyle-related. Changes in VEGF and its receptor act on the placenta, resulting in abnormal blood vessel proliferation and chronic hypoxia. Oxidative stress developed gradually after vascular dysfunction. In the changes of VEGF and its receptors, we also found that nitric oxide (NO) gradually played a crucial role, and VEGF related nitric oxide in pregnant women was found to change in diabetic patients, as shown in Table 2. NO is one of the key contributing factors in the occurrence and development of eclampsia and GDM, and is also associated with endothelial growth factor [58, 59]. The specific mechanism may be related to vascular endothelial oxidative stress [60, 61], and its role is related to the signaling pathway promoting NO production. All of these further stimulate the expression of VEGF and its antagonistic receptors [62], adverse maternal outcomes in GDM are also present in eclampsia [63]. Eclampsia also contributes to GDM development. However, there are significant differences between the two diseases. In the development of GDM, led by VEGF-1 and VEGF165b, the associated subtypes of various VEGF generally increased, while VEGF-related receptors declined [27, 32–35, 37–39]. The associated pathogenesis and progression of eclampsia presented the amount of VEGF and its receptor in the opposite process to GDM [48, 49, 51, 52]. Difference in the number of corresponding antagonistic receptors is the main reason for this. Both diseases are influenced by genetics. Although there is no obvious correlation between the pathogenic genes, both diseases are dominated by genetic factors, and the health status of the mother and the fetus is affected to varying degrees during pregnancy. The detailed mechanism of above aspects of the discussion is shown in Fig. 2. And Fig. 2 specifically illustrates the gradual development of GDM and eclampsia by a variety of VEGF and its receptors, compares the common pathogenic factors of the two diseases, and briefly proposes the treatment methods of the two diseases.In conclusion, the most important mechanism of both GDM and eclampsia lies in the imbalance of VEGF and its receptors, as well as the inhibitory or promoting effect of other related factors. Therefore, it is necessary to timely detect VEGF, the expression of antagonistic receptors and genes, and the value of other related factors in order to effectively monitor the development of GDM and eclampsia. In recent years, the application of nano-biosensors has greatly improved the monitoring sensitivity of VEGF and its receptors. Nano-biosensors are sensitive and reliable tools for the detection of cardiovascular disease and potential cancer risk or early stage cancer [64, 65], Similarly, we can use nano-biosensors to provide timely data for the diagnosis of GDM and eclampsia.

**Table 1:** Relationship between VEGF and its receptor and GDM, PE/Eclampsia

VEGF	The VEGF-R	Country	Year	Association with GDM, PE/eclampsia	Sum up	Quote
VEGF -A	/	Saudi Arabia	2021	In GDM, endothelin-1 and angiogenin-2 were increased with VEGF-A, with no significant increase in G-CSF levels. Angiopoietin-2 was significantly positively correlated with vascular endothelin-1 and VEGF-A, and VEGF-A was significantly positively correlated with endothelin-1 and endothelin-1	VEGF-A is a contributing factor of GDM and is also associated with many other vascular factors. VEGF is not a single factor. Endothelin-1 and colony-stimulating factor in other GDM patients jointly affect vascular health	[27]
/	VEGFR-1 (Flt-1), VEGFR-2 (KD2)	Chile	2017	Lower placental expression levels of Flt-1 (mRNA and protein) are associated with decreased expression of KDR mRNA and increased levels of KDR protein in GDM placenta. In conclusion, GDM is associated with reduced expression of flt-1	Among a variety of receptors, the characteristic expression of GDM is that the low expression of Flt and the high expression of KDR are more significant, and there is a certain influence relationship between the two receptors	[37]
VEGF	VEGFR-1 (Flt-1), VEGFR-2 (KD2)	Brazil	2010	Strong staining of VEGF and VEGFR-2 in vascular and trophoblast cells was limited to trophoblast VEGFR-1; strong staining of VEGFR-1 was observed in vascular cells and trophoblast cells, while VEGF and VEGFR-2 were detected only in trophoblast	The expression of different VEGF and its receptor is different in different parts of GDM patients, which can indirectly infer the pathogenesis of VEGF and its receptor and the pathogenesis of the lesion site	[38]
VEGF	VEGFR-1 (Flt-1), VEGFR-2 (KD2)	Japan	2019	GDM or previous GDM, VEGF-R1 was higher than normal and VEGF-R2 was lower than normal. monocyte chemotactic protein-1 (MCP-1) and eotaxin (eosinophil activated chemokine) have the same expression as VEGF-R	Other pathogenic factors are associated with VEGF receptor to a certain extent, so the pathogenesis of GDM is the result of the combined action of comprehensive factors	[39]
VEGF	/	China	2018	On the basis of relatively controllable risk factors, VEGF rs2146323 and rs3025039 polymorphisms and their expression were significantly associated with GDM risk	Genetic testing before pregnancy to reduce some genetic risk factors besides intuitive risk factors provides effective value for GDM	[40]
VEGF	/	India	2019	Low total values of VEGF 165b, ICAM-1 and AGES in the G DM group,and VEGF was correlated with ICAM-1 and AGEs (glycosylated end products).The GDM and VEGE were associated significantly after correction of AGEs, but the association decreased after correction of ICAM-1	GDM is associated with VEGF ratio (total VEGF165b / VEGF), and V EGF is also associated with ICAM-1 and AGEs, which can determine that ICAM-1 is more important in the mechanism related to VEGF to GDM, and may control and adjust for disease progression	[32]
VEGF	/	Saudi Arabia	2015	It was found that the growth of anti-proliferative VEGF165b subtype in d.HUVEC (human umbilical vein endothelial cells from diabetic mothers) was slower than that in c.HUVEC (human umbilical vein endothelial cells from healthy mothers), and the level of anti-proliferative VEGF165b subtype in d.HUVEC was higher than that in c. HUVEC	The expression of VEGF165b isoform in GDM endothelial cells can be used as one of the serum test criteria; also regulating GDM progression at the nucleotide level, and antagonizing I CAM-1 can balance and adjust patient blood glucose values	[33]
VEGF	/	Austria	2021	GDM patientsplacental tissue lysates were high, and SUCNR1 was positively correlated with vascular endothelial growth factor (VEGF) protein levels in tissue lysates, proving that SUCNR1 was correlated with abnormal placental vascular growth in development	The content of VEGF was determined by checking the content of succinate in the body, from which the condition and development degree of GDM were determined. VEGF can drive the expression of SUCNR1	[34]
VEGF	VEGF-R	Northern Ireland, UK	2021	FKBPL (binding protein),SIRT-1 (deacetylase)and PIGFhave important regulatory functions in the occurrence and progression of GDM. Upregulation of PlGF/VEGF-R1 could be detected in placental serum in gestational patients with type 1 diabetes	The indices of gestational diabetes are changed, and the expression of VEGF-R related protein also changes, other factors FKBPL, SIRT-1 is an important promoting factor, its expression is associated with VEGF-R numerical fluctuations	[35]
VEGF	/	China	2021	In the later stage of GDM progression, VEGF and medium serum RBP4 (retinol-binding protein), Visfatin (lipin) changed, and the above factors are positively associated with fetal weight	The occurrence of GDM is related to the contributing factors, including multiple combined factors; especially the VEGF, its high blood concentration, causing high probability of adverse pregnancy consequences compared	[36]
VEGF	VEGF-R1, VEGF-R2	India	2013	The change in DNA methylation patterns of VEGF, FLT-1 and KDR genes in preeclampsia (PE) indicates that VEGF and its receptors are related to the development of eclampsia	VEGF, flt-1 and KDR promoter regions have several different CpG site methylation, PE related changes, VEGF and receptor value changes as the standard to diagnose PE	[48]
VEGF	sFlt-1	Belgium	2021	Abnormalities in pre-eclamptic VEGF are regulated and affected by post-transcriptional modification of microRNAs (miRNAs), and VEGFmiR-16 is reduced in circulating levels and increased VEGFmiR-200c	VEGF expression is abnormal in PE patients, mir-RNA testing of placental and plasma levels of PE pregnancy found that miR-200C is consistent with placental evidence in placenta, while miR-16 is different. The correlation of the experiment should be verified	[49]
VEGF	/	Iran	2020	Limited to the onset population of Iranian women, the incidence of eclampsia in the population collected expressing the rs10434 gene was also higher than the control group, and initially judged that PE was associated with the specific genotype of VEGF	It is preliminarily judged that V EGF has a correlation with genotype rs10434, but this data is only limited to some female groups, so more reference data are needed to further prove it	[51]
VEGF	/	Britain	2009	In pregnant 12 weeks PE group, normal pregnancy during VEGF165b upward trend, but affected by the pathological influence of PE, its decreasing trend, or increase is given certain delay	Such as mid and late pregnancy detected VEGF165b is lower than normal level, can consider the risk of PE	[52]
VEGF	sFlt-1	China	2019	During PE pathogenesis, VEGF and PIGF decreased decreased. In ELISA test, the values of VEGF and PIGF increased in control group, while sFlt-1 decreased	The expression of VEGF and receptor in PE patients is abnormal, but the influence of detection reagents should be strictly controlled, because different reagents bring different structures	[53]
VEGF	/	Turkey, et al	2020	Pregnant women are at increased risk of pregnancy complications following COVID-19 infection, particularly PE and GDM. Placental damage and vascular embolism leading to hypoxia were the main induction of VEGF imbalance(In COVID-19 groups), and the negative correlation between neutrophils and VEGF-A	COVID 19 is a complication of pregnancy, and indirect evidence suggests that the virus exacerbates VEGF imbalance, Further inferentially, COVID-19 may also be promote or exacerbate the effects of pregnancy complications	[54–56]

**Table 2:** NO impacted in GDM,PE/ Eclampsia and its association in this regard with VEGF

	Years	NO	VEGF/VEGF-R	GDM/ Eclampsia/PE	Supplement	Cite
1	2016	↑ (Availability of NO decreased)	VEGF	GDM	The change of NO may be related to oxidative stress mechanism. May be related to post-transcriptional and translational NO synthase (NOS) regulation, including phosphorylation/dephosphorylation cycles	[60]
		↓	VEGF	PE	Same as above	
2	2012	↑	VEGF	GDM	Altered endothelial L-arginine/NO signaling pathway	[61]
		↓		PE	Arginine deficiency recorded in the pre-placenta leads to a decrease in NO and an increase in superoxide formation	
3	2011	unclear	VEGF	GDM	NO regulates the vascularization of pregnancy complications such as GDM and PE	[71]
			VEGF-R	PE	Same as above	
4	2017	↑	VEGF	GDM	L-arginine/NO signaling pathway	[72]
5	2019	↓	VEGF-R(sFlt-1)	PE	There was a certain correlation between NO,sFlt-1 and BNP	[58]
6	2015	↓ (severe) ↓↓ (mild)	PLGF	PE	The changes of PLGF and NO levels in groups may be closely related to the pathogenesis and development of early-onset PE	[59]
7	2009	↓	VEGF	PE	The expression of nitric oxide in patients with eclampsia was higher than that in patients with preeclampsia	[73]
		↓↓	VEGF	Eclampsia	Same as above

### GDM and eclampsia are related to VEGF treatment

In view of the above experiments and case studies demonstrating the important role of VEGF in disease progression, related treatments for VEGF imbalance have been used. These can also indirectly show that GDM/eclampsia therapy has a strong correlation with VEGF. Many commonly used therapeutic drugs inhibit vascular endothelial growth by regulating VEGF levels. Among them, the most important treatment method for GDM is insulin treatment. Insulin does not pass through the placenta and thus has no effect on the fetus's growth and development. Magnesium sulfate is the most commonly used treatment drug for eclampsia, with other antihypertensive drugs also included in the alternative category. However, when considering the impact of VEGF, the treatment and prevention of GDM and eclampsia can be done in other ways. According to some studies have shown that aspirin is a preferable drug because low-dose aspirin acts as an antiplatelet production agent [66, 67]. Besides playing an important cardiovascular role, it can also prevent and delay the development of PE and GDM, especially in those possessing the underlying genes. In pregnant women with multiple high-risk factors, aspirin dipyridamole can be used in moderation to effectively prevent the occurrence of disease. It can also reduce the risk of related diseases in other organs. However, as it is a non-steroidal anti-inflammatory drug, it can also lead to the emergence of gastrointestinal diseases. As a result, in the elderly patients with simultaneous gastrointestinal diseases or poor function of various organs, Vitamin D may be considered for alternative therapy [68, 69]. Reducing disease and the risk of gastrointestinal bleeding are important considerations, alongside cost effectiveness.

In addition, pravastatin is another important treatment method for PE [53]. Pravastatin can effectively regulate PLGF and reduce sFlt-1 levels to improve hypertension, proteinuria, and other diseases. However, special attention should also be paid to its role in liver and kidney metabolism. The effectiveness of the natural compound Vitexin can also be considered to reduce the onset of PE by inhibiting HIF-1 (acylase)/VEGF [70]. Melatonin is also an Indole heterocyclic compounds that has been shown to be effective in regulating inflammatory factors in diabetic retina [67], which can reduce VEGF expression. It may also play an important role in the treatment of GDM, although this requires further verification. Treatment with the above drugs should consider taking pregnant women's and fetuses' growth and development into account. Additional clinical trials are required to ensure that harmful side effects are minimized.

## Limitations

This review still has many limitations. On the one hand, there are still some mechanisms in the process of writing the article based on personal summary and discussion of the literature, and all the findings are based on personal views, but it is still subjective to some extent. On the other hand, the most of the references and materials in this section are based on about ten years of content, so there may be time limitation.

## Conclusions

This review focuses on the strong association between VEGF and its receptor and gestational diabetes and the progression of PE-eclampsia. First of all, we learned that VEGF and its receptor have changed in both diseases, but the specific value expression is different. In the progression of GDM, the value of various types of VEGF increases while its receptor decreases, whereas VEGF receptor is significantly increased in eclampsia patients. Second, endothelin and other factors are involved in both diseases. It indicates that the changes brought about by the two diseases are influenced by a variety of factors. Finally, although the expression of special genetic factors differs between the two diseases, the gene expression of VEGF is greatly increased in both, indicating that genetic factors are an important factor of pregnancy diseases. Through the research summarized in this review, we can improve the changes in the values of VEGF, its receptors, and related factors. Understanding the progression of GDM and gestational eclampsia is crucial for a timely response to disease risk. Monitoring genotype and serum data can aid in predicting disease stages, from high-risk to diagnosis and treatment, to ultimately reduce adverse effects before and during the pathogenesis process. In this study, people will have a better understanding of gestational diabetes and eclampsia in the future, which can reduce the potential risk of the disease. Some of the effects of VEGF and its receptors on the human body are irreversible. If VEGF and corresponding receptors can be timely balanced at an early stage, the risk of pregnancy complication can be reduced in the future.

## Data Availability

Not applicable.
